# Single-Trial Sparse Representation-Based Approach for VEP Extraction

**DOI:** 10.1155/2016/8569129

**Published:** 2016-10-11

**Authors:** Nannan Yu, Funian Hu, Dexuan Zou, Qisheng Ding, Hanbing Lu

**Affiliations:** ^1^School of Electrical Engineering and Automation, Jiangsu Normal University, Xuzhou 221116, China; ^2^Department of Internal Neurology, Xuzhou Central Hospital, Xuzhou 221116, China

## Abstract

Sparse representation is a powerful tool in signal denoising, and visual evoked potentials (VEPs) have been proven to have strong sparsity over an appropriate dictionary. Inspired by this idea, we present in this paper a novel sparse representation-based approach to solving the VEP extraction problem. The extraction process is performed in three stages. First, instead of using the mixed signals containing the electroencephalogram (EEG) and VEPs, we utilise an EEG from a previous trial, which did not contain VEPs, to identify the parameters of the EEG autoregressive (AR) model. Second, instead of the moving average (MA) model, sparse representation is used to model the VEPs in the autoregressive-moving average (ARMA) model. Finally, we calculate the sparse coefficients and derive VEPs by using the AR model. Next, we tested the performance of the proposed algorithm with synthetic and real data, after which we compared the results with that of an AR model with exogenous input modelling and a mixed overcomplete dictionary-based sparse component decomposition method. Utilising the synthetic data, the algorithms are then employed to estimate the latencies of P100 of the VEPs corrupted by added simulated EEG at different signal-to-noise ratio (SNR) values. The validations demonstrate that our method can well preserve the details of the VEPs for latency estimation, even in low SNR environments.

## 1. Introduction

Evoked potentials (EPs) are bioelectrical signals that are generated by the central nervous system when the latter is stimulated by well-defined external stimuli. Depending on the modality of stimulation, EPs are categorised into auditory evoked potential (AEP), visual evoked potential (VEP), and somatosensory evoked potential (SEP). In clinical environments, these signals are used to reflect the various functions of auditory, optic, and sensory nerve sense-conducting pathways. In this paper, we concentrate on the second type, namely, the VEPs. Generally speaking, there exist three prominent components (N75, P100, and N145) in the VEP signal, whereas the preceding and following segments are almost flat. Of the three components, the P100 wave is the most significant and stable; hence, it is the most important component in clinical applications [1].

VEP signals have time-locked (quasiperiodic) characteristics and are always accompanied by ongoing electroencephalogram (EEG) signals. Moreover, the signal-to-noise ratio (SNR) of VEP records is usually low (−5 to −10 dB). Ensemble averaging (EA) is the most widely used method for estimating VEP against a noisy background. However, EA cannot be used to detect latency and amplitude variations from one trial to another; thus, single-trial analysis is better suited for investigations into the dynamics of brain activation. The single-trial VEP estimation is very meaningful in cognitive science research and clinical applications, such as brain-computer interfacing and intraoperative monitoring [2].

Many single-trial EP estimation methods have been proposed over the past two decades. These methods can be divided into two categories, namely, denoising methods and separation methods. The denoising methods assume that the measurement of the VEP is corrupted by noise and that the main source of noise is the EEG. Many conventional denoising methods have been applied, such as the Wiener filter [3], Kalman filter [4], and ARX [5]. Among these methods, ARX is widely recognised and has previously been applied to monitor the depth of anaesthesia during surgery. In ARX, the EEG can be viewed as an autoregressive (AR) model driven by white noise, and the EP can be modelled by an ARMA filter with a known signal accurately. The known signal is typically the average of the reference EPs (AREP). The orders and parameters of the AR and ARMA models can be estimated by utilising various optimisation techniques, such as the final prediction error (FPE) [6] and the least-squares (LS) method [7]. The EPs can then be reconstructed by ARMA filtering with the AREP. Recently, Cerutti et al. [6] found that EP extraction using ARX modelling is only capable of extracting latency EP variations in relatively high SNRs and that it is completely invalid because the latency varies greatly compared with the AREP from systemic experiments. The separation methods separate the VEP and EEG signals by modelling them based on their characteristics, such as wavelet transformation and sparse representation.

Meanwhile, Causevic et al. [8] and Martazi et al. [9] used wavelet transformation to separate the EP and EEG signals. Sparse coding is a powerful tool in analysing nonstationary signals, and it has shown significant success in signal denoising and separation. Xu and Yao [10] proposed the mixed overcomplete dictionary-based sparse component decomposition method (MOSCA), which decomposes the EP and EEG signals in the wavelet dictionary and discrete cosine transform (DCT) dictionary, respectively. However, given that EEG is not considered white noise and that many components of EP and EEG look alike in a single trial, their components are represented by the wrong dictionaries and their corresponding coefficients. Therefore, MOSCA cannot separate the EP and EEG signals sufficiently [11, 12].

In this paper, we present a novel sparse representation-based approach to solving the VEP extraction problem. Instead, of the mixed signals from the EEG and EP, we utilised an EEG in a previous trial that did not contain VEP to identify the parameters of the EEG AR model. Then, we used sparse representation in the ARMA model, instead of MA, to simulate the VEP. The sparse coefficients can be calculated by an optimisation method. Finally, the VEP can be derived from the AR model. Experiments carried out on synthetic and real data confirm the superior performance of our method. The rest of the paper is organised as follows.  Section 2 provides the details of our single-trial estimation algorithm.  Section 3 contains experimental results obtained from the proposed method and a comparison with ARX and MOSCA.  Section 4 provides the conclusions.

## 2. Method

Let the VEP signal *p*(*k*) ∈ *R* ^*N*×1^ to be estimated be corrupted by noise from ongoing background activities. The main source of noise is the spontaneous EEG *e*(*k*) ∈ *R* ^*N*×1^. The measurement *s*(*k*) ∈ *R* ^*N*×1^ is given by

(1) $$\begin{array}{c} s\left(\;k\;\right)=p\left(\;k\;\right)+e\left(\;k\;\right). \end{array}$$

We need to design a method that can remove the noise from *s*(*k*), getting as close as possible to the original EP signal *p*(*k*) [13].

### 2.1. The VEP Signal

In ARX, VEP *p*(*k*) is derived by filtering the reference *u*(*k*) ∈ *R* ^*N*×1^, which is chosen to be the average of a sufficient number of trials and can represent the general form of the evoked response under analysis, by the ARMA model parameters; that is,

(2) $$\begin{array}{c} \hat{P}\left(\;z\;\right)=\left(\;\frac{B\left(z^{-1}\right)}{A\left(z^{-1}\right)}\;\right)U\left(\;z\;\right), \end{array}$$

where $\hat{P}(z)$ and *U*(*z*) are the *z*-transform of $\hat{p}(k)$ and *u*(*k*) and *A*(*z* ^−1^) = 1 − ∑_*i*=1_^*n*^ *a*_*i*_ *z* ^−*i*^, *B*(*z* ^−1^) = *z* ^−*d*^∑_*j*=0_^*m*−1^ *b*_*j*_ *z* ^−*j*^. Sparse coding is a powerful tool for the analysis of nonstationary signals; it has achieved significant success in signal denoising and separation. Compared with ARMA, sparse coding is more flexible and uses the dictionary and the corresponding coefficient to represent signals. VEP has been proven to have strong sparsity over an appropriate dictionary in our previous paper [12]. Thus, in the current paper, we use sparse coding to represent the single-trial VEP instead of the MA model in ARMA. Therefore, formula (2) can be rewritten as

(3) $$\begin{array}{c} \hat{p}\left(\;k\;\right)=\frac{B\left(z^{-1}\right)u\left(k\right)}{A\left(z^{-1}\right)}=\frac{G\theta }{A\left(z^{-1}\right)}, \end{array}$$

where *G* ∈ *R* ^*N*×*M*^ and *θ* ∈ *R* ^*M*×1^ are the dictionary and sparse coefficient of *B*(*z* ^−1^)*u*(*k*), respectively. The transfer function *B*(*z* ^−1^)/*A*(*z* ^−1^) merely represents a mechanism to incorporate deterministic VEP *p*(*k*) variations into the reference signal *u*(*k*), rather than a physiologically meaningful process.

### 2.2. Dictionary Construction

Inspired by the modelling method in [14], we proposed a dictionary construction method for the EP signal, as reported in our previous paper. This method assumes that the atoms in the dictionary can be extracted from a reference signal and that the single-trial EP can be decomposed sparsely by the dictionary. Many previous experiments have demonstrated this result.

The reference signal *u*(*k*) consists of a superposition of *M* components expressed as

(4) $$\begin{array}{c} u\left(\;k\;\right)=\overset{M}{\underset{m=1}{\sum }}a_{m}s_{m}\left(\;k\;\right). \end{array}$$

*u*(*k*) can be acquired by AREP. *s*_*m*_(*k*) can be extracted from *u*(*k*) using a certain filtering window function, such as Hamming window and Blackman window. The central location and width of the window are determined by the location of point of peak (and valley) amplitude and peak (and valley) width of the *m*th component. The dictionary can be represented by

(5) $$\begin{array}{c} D=\left(\begin{array}{cccc} S_{1} & S_{2} & \cdots & S_{M} \end{array}\right), \end{array}$$

where  *S*_*m*_ ∈ *R* ^*N*×2*d*^, and

(6) SmT=smd⋯⋯smN⋯0⋮⋮⋮⋮⋮⋮sm2sm3⋯⋯smN0sm1sm2sm3⋯⋯smN0sm1sm2sm3⋯smN−1⋮⋮⋮⋮⋮⋮0⋯0sm1⋯smN−d.

Then, *u*(*k*) can be represented by the dictionary *D* and the coefficient *θ*_1_, as

(7) $$\begin{array}{c} u\left(\;k\;\right)=D\theta_{1}. \end{array}$$

In this paper, we aim to construct the dictionary *B*(*z* ^−1^)*u*(*k*). The transfer function *B*(*z* ^−1^) represents a mechanism, which incorporates deterministic single-trial EP variations into the reference signal, rather than a physiologically meaningful process. From this, it follows that

(8) $$\begin{array}{c} B\left(\;z^{-1}\;\right)u\left(\;k\;\right)=\overset{m+d-1}{\underset{l=0}{\sum }}b_{l}u\left(\;k-l\;\right), \end{array}$$

where  *m*  and  *d*  are usually small positive integers. Given that *u*(*k*) is sparse on dictionary *D*, *B*(*z* ^−1^)*u*(*k*) is also sparse on dictionary *D*. Thus, in this paper, *G* = *D*.

### 2.3. EEG Signal

Similar to ARX, in this paper, the EEG *e*(*k*) is viewed as an AR model driven by white noise *w*(*k*); that is,

(9) $$\begin{array}{c} \hat{e}\left(\;k\;\right)=\frac{1}{A\left(z^{-1}\right)}w\left(\;k\;\right). \end{array}$$

The parameters can be estimated using the least-squares method. We assume that the statistical characteristics of the EEG in the successive trials are similar, as has been reported in many papers [7, 8]. Thus, in the current paper, instead of the mixed signal of EEG and EP, we utilise the EEG from a previous trial, to estimate the parameters of AR model. This EEG does not contain EP.

### 2.4. Single-Trial Extraction

Substituting formulas (3) and (5) into formula (1), we get

(10) $$\begin{array}{c} \hat{s}\left(\;k\;\right)=\hat{p}\left(\;k\;\right)+\hat{e}\left(\;k\;\right)=\frac{G\theta }{A\left(z^{-1}\right)}+\frac{1}{A\left(z^{-1}\right)}w\left(\;k\;\right). \end{array}$$

Then,

(11) $$\begin{array}{c} A\left(\;z^{-1}\;\right)\hat{s}\left(\;k\;\right)=G\theta +w\left(\;k\;\right). \end{array}$$

Let $x(k)=A(z^{-1})\hat{s}(k)$; then, formula (11) can be simplified as

(12) $$\begin{array}{c} x\left(\;k\;\right)=G\theta +w\left(\;k\;\right). \end{array}$$

Hence,

(13) θ^=arg⁡minθ θ0s.t. xk−G·θ2≤ε0,

where *ε*_0_ is determined by the variance of the EEG. Formula (10) can be solved by using optimisation methods, such as basis pursuit (BP) [15], orthonormal matching pursuit (OMP) [16], and Lasso [17].

The single-trial VEP can then be reconstructed by using

(14) $$\begin{array}{c} \hat{p}\left(\;k\;\right)=\frac{G\hat{\theta }}{A\left(z^{-1}\right)}. \end{array}$$

## 3. Experimental Results

### 3.1. Analysis of the Simulations

Computer simulation is conducted to verify the performance of our proposed VEP signal extraction method. Depending on the characteristics of the VEP, the simulated VEP is constructed with three components and is expressed as

(15) pk=3exp⁡−k−752202−7exp⁡−k−100+m2152+1.2exp⁡−k−1452152.

**Figure 1:**
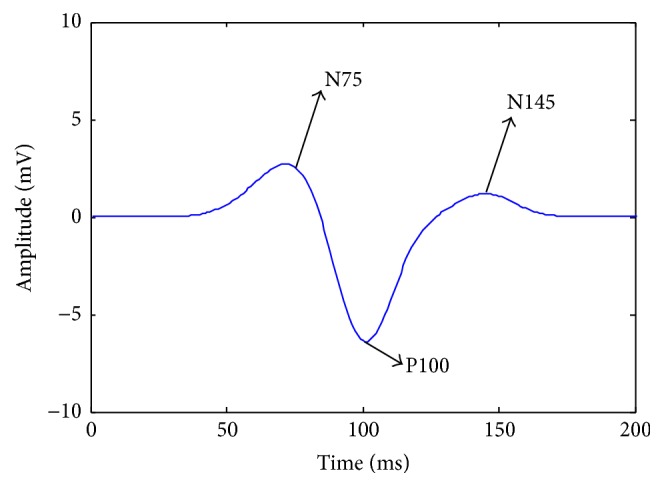
Simulated EP indicating three components: N75, P100, and N145.

The three Gaussian functions represent a prominent VEP with similar morphological characteristics to those of the negative (N75), positive (P100), and negative (N145) peaks of a real VEP, respectively. The simulated VEP is shown in Figure 1.

The background EEG that is superimposed on the EP signal is simulated by an AR process [18], which is given by

(16) ek=1.5084ek−1−0.1587ek−2−0.3109ek−3−0.0510ek−4+wk,

where *w*(*t*) is the Gaussian white noise. The simulated VEP is shown in Figure 1.

In this paper, we assume that the AR parameters of spontaneous EEG in two consecutive trials are extremely similar. In order to validate this assumption, three consecutive trials of spontaneous EEG signals *e*_*i*_(*k*)  (*i* = 1,2, 3) are chosen randomly for the experiment. We compute their least-squares AR model with an approach. We set

(17) A1z−1=1−3.106z−1+4.291z−2−3.465z−3+1.683z−4−0.389z−5,A2z−1=1−3.138z−1+4.404z−2−3.621z−3+1.759z−4−0.399z−5,A3z−1=1−3.143z−1+4.504z−2−3.887z−3+1.643z−4−0.410z−5.

Then, each *A*_*i*_(*z* ^−1^) is used to transform the EEG signal in the other trial, so these parameters are, respectively, changed as

(18) A1′z−1=A3z−1,A2′z−1=A1z−1,A3′z−1=A2z−1.

**Figure 2:**
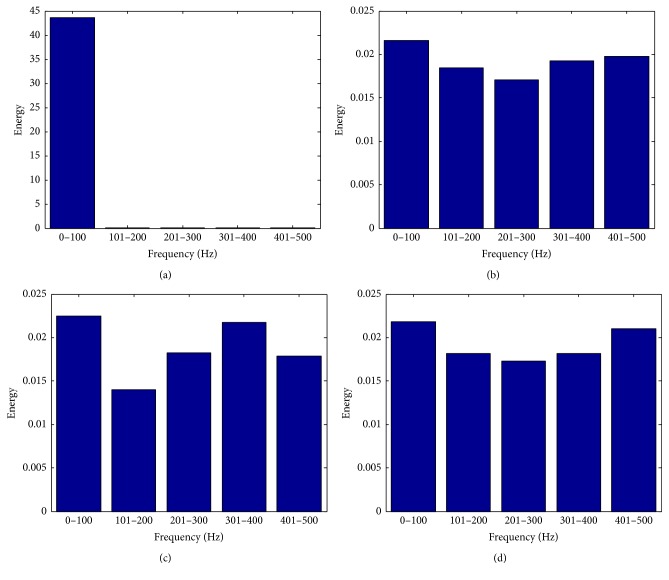
The frequency band energy spectrum. (a) EEG *e*_1_(*k*); (b) *w*_1_(*k*); (c) *w*_2_(*k*); (d) *w*_3_(*k*).

**Figure 3:**
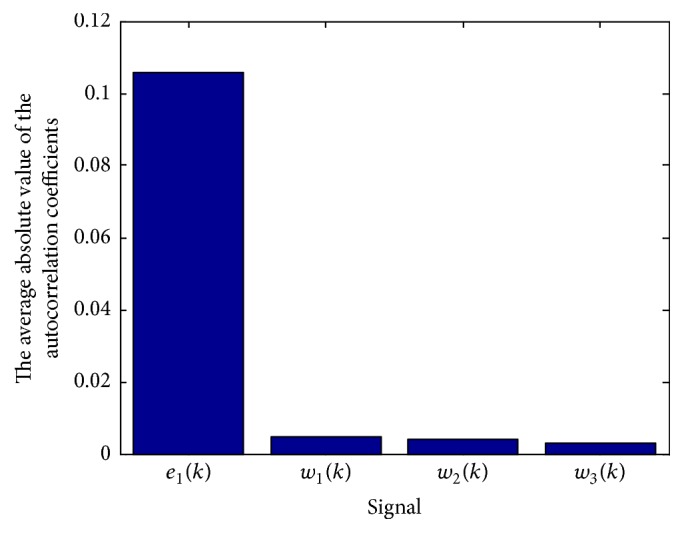
The average absolute value of the autocorrelation coefficients.

The three EEG signals are transformed by *A*_*i*_′(*z* ^−1^); that is, *W*_*i*_(*z*) = *A*_*i*_′(*z* ^−1^)*E*_*i*_(*z*), where *W*_*i*_(*z*) and *E*_*i*_(*z*) are the *z*-transform of *w*_*i*_(*k*) and *e*_*i*_(*k*), respectively. In Figures 2 and 3, we, respectively, provide the frequency content and independence of *w*_*i*_(*k*). As can be seen in the figures, compared with *e*_1_(*k*), the energy of each frequency band of *w*_*i*_(*k*) is more uniform, and the autocorrelation coefficients of *w*_*i*_(*k*) are lower.

**Figure 4:**
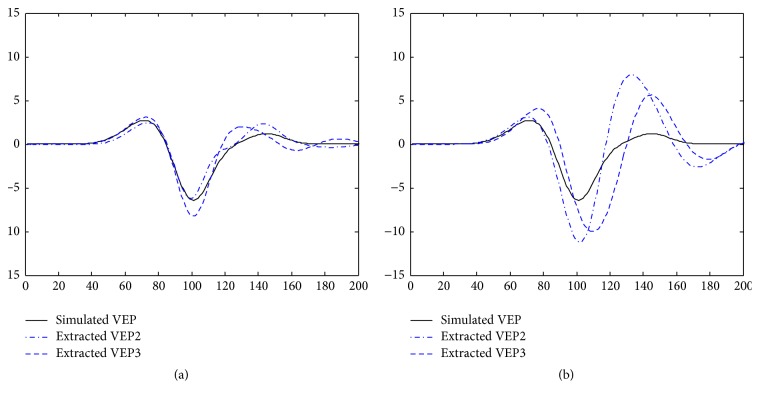
The VEP2 and VEP3 extracted with different SNR values. (a) SNR = −5 dB; (b) SNR = −10 dB.

Let *A*_*i*_ ∈ *R* ^6×1^ represent the parameter vectors of *A*_*i*_(*z* ^−1^) and *d*_*ij*_ = [∑_*l*=1_^6^((*A*_*il*_ − *A*_*jl*_)/*A*_*jl*_)^2^]^1/2^ represent the difference between *A*_*i*_ and *A*_*j*_. From formula (13), we obtain *d*_12_ = 0.0743 and *d*_13_ = 0.1626. In order to test the robustness of the proposed method in case inaccurate estimations of the AR coefficients are obtained, we use *e*_1_(*k*) to estimate the AR parameter and *e*_2_(*k*) and *e*_3_(*k*) to generate the measurements *s*_2_(*k*) and *s*_3_(*k*), respectively. Then, the extracted VEP2 and VEP3 from *s*_2_(*k*) and *s*_3_(*k*) with our proposed method are shown in Figure 4. With our method, the VEP2 and VEP3 are extracted from *s*_2_(*k*) and *s*_3_(*k*). As shown in the figure, when SNR = −5 dB, both VEP2 and VEP3 show results that approach the simulated VEP.

Table 1 shows the mean and standard deviation of SNR obtained from 100 extracted VEP2 or VEP3. As shown in the table, with the same SNR, the SNR values of VEP2 and VEP3 are similar, although *d*_13_ is two times larger than *d*_12_.

During estimation, the observed SNR values may change over time due to the nonstationary characteristics of the EEG. Therefore, in this experiment, the performance of our method is examined under various SNR conditions. The EEGs are generated with formula (16).

**Figure 5:**
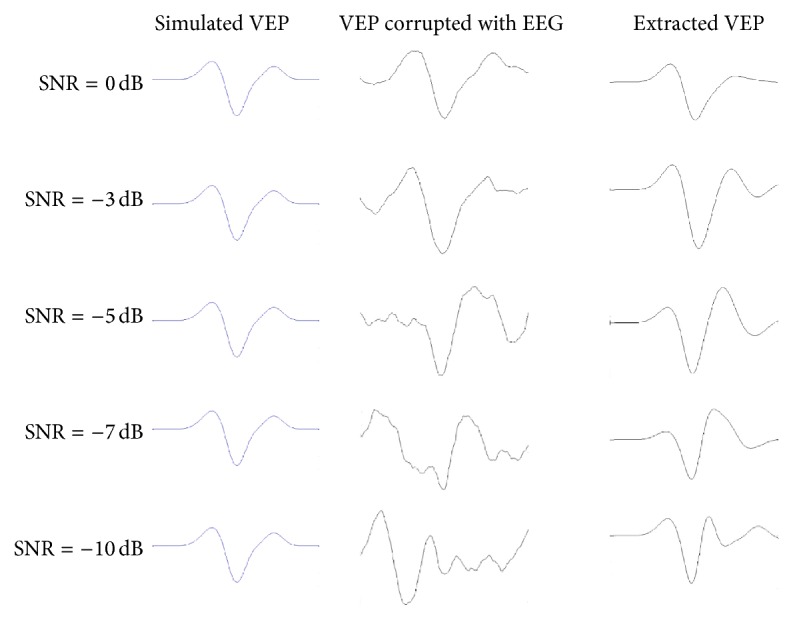
Single-trial VEP extracted by our method with different SNR values.

As shown in Figure 5, although the estimation performance degrades with decreasing SNR, the prominent morphological characteristics (N75, P100, and N145) are preserved in all the SNR values.

**Figure 6:**
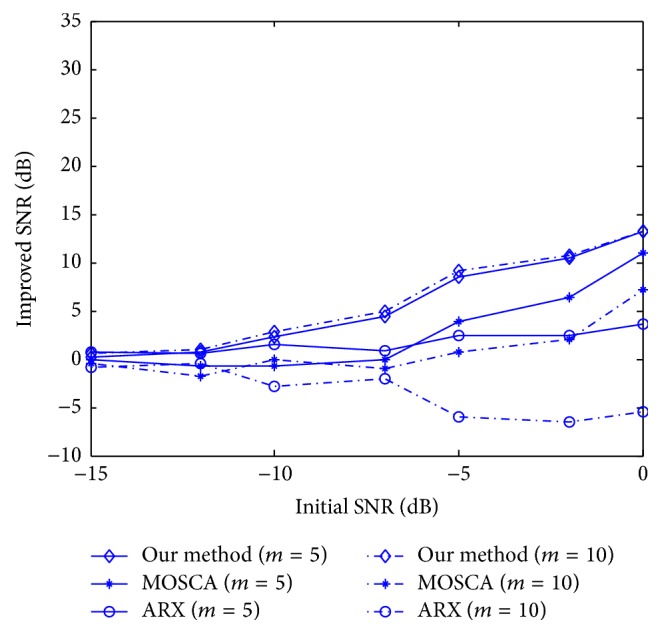
The improved SNR of VEP with different latencies of P100 using our method, MOSCA, and ARX.

The average values of SNR obtained with our method, MOSCA [10], and ARX [6] are shown in Figure 6. The results are acquired by identifying the average of 100 trials for each piece of data. In our method, the dictionary of VEP is constructed by using formula (15) where *m* = 0. Similarly, in ARX, the reference VEP is generated by formula (15) where *m* = 0. In this experiment, we change the latency of P100 by setting *m* = 5 and *m* = 10. We can see from this figure that our method consistently demonstrates the greatest improvement in all methods. Compared with sparse coding, ARX (*m* = 5) shows superior performance at low initial SNRs. However, when the latencies change greatly (*m* = 10), ARX method degrades seriously. We can also see that the latency change has hardly any impact on the estimation performance of our method.

To increase the objectivity of the evaluation, for each SNR and *m*, we generate data from 50 trials and then estimate the latencies of P100. As shown in Table 2, we change *m* from −10 to 10 and the SNR from −10 dB to 0 dB and estimate the single-trial VEP signal. Results show that, with the decrease of SNR, all standard deviations also increase. The RMSE value depends primarily on the SNR, rather than on the variations of latency (*m*), thereby indicating that our method is appropriate for tracking the latency variations when SNR ≥ −10 dB.

### 3.2. Analysis of the Real VEP

To further evaluate the performance of our method, we collected VEPs from three pairs of eyes of three human subjects during pattern reversal VEP experiments. The basic data obtained from the three subjects are shown in Table 3.

**Figure 7:**
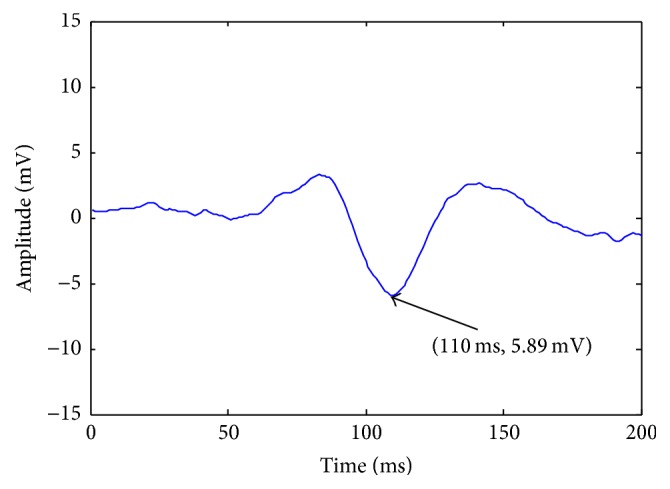
The average VEP.

An example from the 50 trials is selected randomly from the original recorded VEPs of subject 2's right eye.  Figure 7 shows the corresponding average VEP.

**Figure 8:**
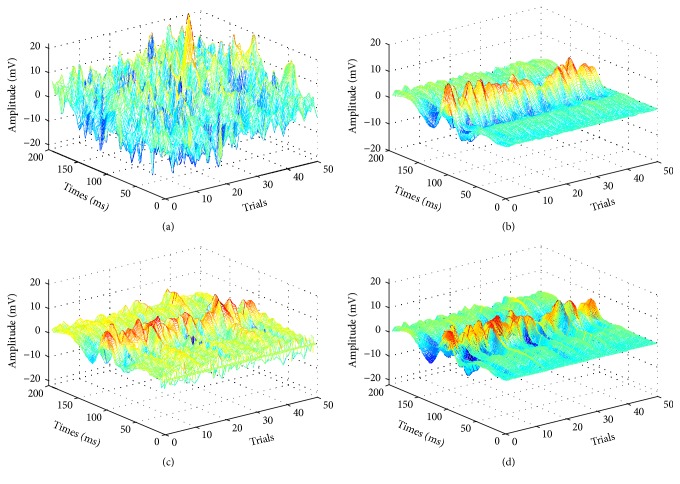
(a) The original 50 VEP signals. (b) The estimated VEP with our method. (c) The estimated VEP with MOSCA. (d) The estimated VEP with ARX.

We extract the VEPs with our method, MOSCA, and ARX, and the results are shown in Figure 8. Clearly, the three components N75, P100, and N145 of VEPs extracted with our method are prominent, and the signals estimated using our method are more similar to the ensemble average signal.

**Figure 9:**
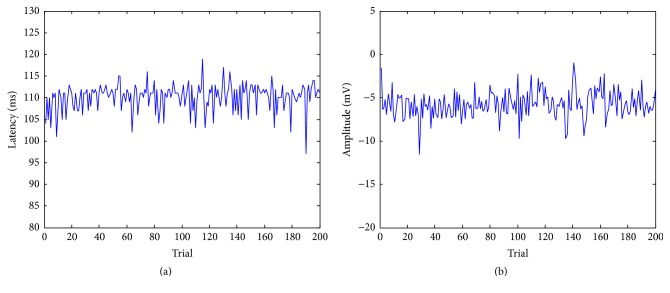
(a) The estimation of amplitudes of P100 of 200 trials. The mean is 110.16 ms and the standard deviation is 3.07 ms. (b) The estimation of latencies of P100 of 200 trials. The mean is −5.73 mv and the standard deviation is 0.54 mv.

Then, our method is used to estimate the amplitudes and latencies of P100 in 200 trials. As shown in Figure 9, the variations in amplitudes and latencies are significant, whereas most amplitudes are between −7 and −4 and most latencies are between 100 and 115. These results have good agreement with those observed in practice.

## 4. Conclusions

Single-trial EP estimation is a very useful tool in cognitive science-related studies and clinical applications. Many investigations have been carried out and some amount of success has been achieved. However, only a few practical methods have been proposed. ARX modelling is a classical method that has been applied in clinical practice for several years. However, this method has limitations regarding the tracking of latency variations and is only capable of extracting latency variations of an EP under relatively high SNR values. Meanwhile, sparse coding is a powerful tool in signal denoising, and EPs have been proven to have strong sparsity over an appropriate dictionary. Inspired by this idea, in this paper, we introduce sparse coding into the ARX model and propose a novel single-trial VEP extraction method based on ARX and sparse coding. Compared with ARMA, sparse coding is more flexible. It uses the best matching atoms from the dictionary to represent the EP signal without needing to estimate the number of atoms beforehand. By transforming the electroencephalography signal into white noise, the single-trial EP estimation is transformed into a signal denoising problem for white noise. With the dictionary constructed specially for EPs, the EP signal can be extracted easily with sparse coding. Moreover, since the location of the atom in the dictionary has no influence on the effectiveness of sparse decomposition, variations of the amplitude and latency of EPs have only a minor impact on the performance of the proposed method. The proposed method can thus track EP signal variations. We conducted a series of experiments on synthetic and real data, and the results have been evaluated using waveform observation and several metrics. The validations demonstrate that our method can well preserve the EP details of latency and amplitude estimation simultaneously, even under low SNR conditions.

## Figures and Tables

**Table 1:** The SNR of VEP2 and VEP3 extracted by our method.

SNR (dB)	VEP2		VEP3	
	Mean	Standard deviation	Mean	Standard deviation
−5	9.98	0.07	9.47	0.08
−10	5.48	0.24	5.24	0.17

**Table 2:** Latency of P100 extracted by our method.

*m*	SNR (dB)							
	0		−3		−5		−10	
	Mean	Standard deviation	Mean	Standard deviation	Mean	Standard deviation	Mean	Standard deviation
−10	90.8 ms	1.4 ms	90.4 ms	3.2 ms	89.2 ms	5.8 ms	91.1 ms	6.2 ms
−5	94.9 ms	1.4 ms	95.5 ms	3.9 ms	94.5 ms	5.9 ms	95.7 ms	6.6 ms
0	100.8 ms	1.8 ms	100.7 ms	3.4 ms	102.8 ms	4.2 ms	101.3 ms	6.2 ms
5	104.7 ms	1.5 ms	105.2 ms	3.8 ms	103.4 ms	5.7 ms	104.7 ms	6.4 ms
10	110.7 ms	1.9 ms	110.2 ms	3.9 ms	112.4 ms	6.1 ms	112.4 ms	7.4 ms

**Table 3:** The basic data of 3 subjects.

Subject	Age	Sex	Vision
1	25	F	Normal
2	24	M	Normal
3	25	F	Normal
